# Alternative Carrier Solvents for Pigments Extracted from Spalting Fungi

**DOI:** 10.3390/ma11060897

**Published:** 2018-05-27

**Authors:** Lauren Pittis, Diego Rodrigues de Oliveira, Sarath M. Vega Gutierrez, Seri C. Robinson

**Affiliations:** 1 Department of Botany and Plant Pathology, Oregon State University, Corvallis, OR 97331, USA; lpittis@gmail.com; 2 Wood Science & Engineering, Oregon State University, Corvallis, OR 97331, USA; diego.rodrigues@oregonstate.edu (D.R.d.O.); Sarath.vega@oregonstate.edu (S.M.V.G.)

**Keywords:** spalting, fungal pigments, solvents, wood coloration, *Chlorociboria aeruginosa*, *Scytalidium cuboideum*

## Abstract

The use of both naturally occurring and synthetic pigmented wood has been prevalent in woodcraft for centuries. Modern manifestations generally involve either woodworkers’ aniline dyes, or pigments derived from a special class of fungi known as spalting fungi. While fungal pigments are more renewable than anilines and pose less of an environmental risk, the carrier required for these pigments—dichloromethane (DCM)—is both problematic for humans and tends to only deposit the pigments on the surface of wood instead of evenly within the material. Internal coloration of wood is key to adoption of a pigmenting system by woodworkers. To address this issue, five solvents that had moderate solubility with the pigments extracted from *Chlorociboria aeruginosa* and *Scytalidium cuboideum* were identified, in the hopes that a reduction in solubility would result in a greater amount of the pigment deposited inside the wood. Of the tested solvents, acetonitrile was found to produce the highest internal color in ash, Douglas-fir, madrone, mountain hemlock, Port-Orford cedar, Pacific silver fir, red alder and sugar maple. While these carrier solvents are not ideal for extracting the pigments from the fungi, acetonitrile in particular does appear to allow for more pigment to be deposited within wood. The use of acetonitrile over DCM offers new opportunities for possible industrial spalting applications, in which larger pieces of wood could be uniformly pigmented and sold to the end user in larger quantities than are currently available with spalted wood.

## 1. Introduction

Spalting is the term used for any coloration of wood caused by fungi [1,2]. This coloration can vary according to the type of wood decay. White rot fungi bleaches wood, as their enzymes remove primary lignin [3,4]. These fungi can also create zone lines (mostly composed of melanin) as a result of somatic incompatibly [5,6] or substrate desiccation [7]. Within the decay fungi there are also pigmenting fungi [1]. This group produces secondary metabolites that can color wood. Most are Ascomycetes [8]. These fungi use their pigments as a method of resource capture when competing against other fungi on decaying wood [9]. This feature is used for laboratory pigment production, as the inclusion of sterilized, white-rotted wood into growing media can stimulate pigment production in spalting fungi [10]. The most well-known pigmenting fungi (in terms of woodworking) are *Chlorociboria* spp. [11,12,13], as their secondary metabolite, xylindein, pigments wood in blue-green shades [9,14,15,16,17,18]. Another well-known spalting fungus is *Scytalidium cuboideum*, which makes a red pigment called draconin-red [19]. This pigment was first described in 1995 as a naphthoquinone [20] (xylindein is also a naphthoquinone). Recent studies on draconin-red suggested a crystalline configuration [21,22], which was confirmed with further crystallographic studies [23].

Spalting pigments have been used as colorants on diverse materials such as wood [24,25,26], bamboo [27] and textiles [19,28,29,30], however they are most commonly used for wood turnings.

**Figure 1:**
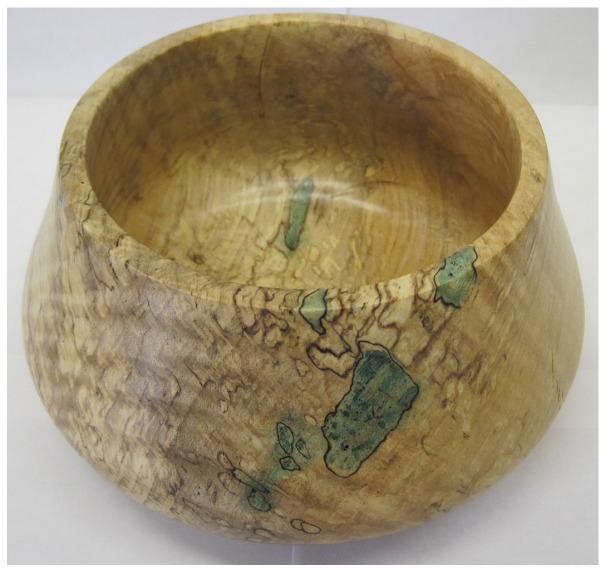
Turned maple with zone lines, and pigment from *Chlorociboria* spp. Source: Seri C. Robinson.

Spalted wood is commonly used throughout industrial and artistic woodworking [31]. An example of a turned bowl made from spalted wood (zone lines, white rot, and the blue-green pigment from *Chlorociboria* species) is shown in Figure 1. The history of spalted wood began as an artistic endeavor in the 14th century [11,12,13], and is used for both woodworking/woodturning and various scientific applications today, such as thin films for solar cells [32], paint colorants [33], and textile dyes [19,28,29]. Most modern spalted woodworking focuses on using collected spalted wood [34] or inoculating wood with pigmenting fungi to produce a specific range of colors [35].

A study by Robinson et al. (2014) developed a method for extracting spalting fungal pigments from wood and reapplying them in a controlled manner back into sound wood in order to speed up the spalting process [35]. This method established that dichloromethane (DCM) was the best solvent for the extraction and carrying of the pigments and reduced the timeframe for controlled spalting (where the fungi are introduced to the wood to control the color output) from months to hours. The DCM-solubilized pigments moved readily through the wood and pigmented both ends of any given piece, but did not, unfortunately, leave any color on the inside of the wood [25,26]. Hence, this new technique was appropriate for surface coloration, but not for coloring pieces that would later be machined down. The authors hypothesized that no interior coloration occurred because pigments had more solubility with DCM than the wood and hence the binding of the pigment only occurred where the DCM evaporated This is in contrast to aniline dyes, which have a long history of use by woodworkers, as these dyes readily pigment the exterior and interior of wood. Unfortunately, aniline dyes are not light stable, even in indoor light, and are known to be toxic to humans [36]. Due to the unique characteristics of spalting pigments, such as UV-light resistance and hydrophobicity [37], there is considerable interest in replacing commonly used aniline dyes with these pigments, as well as mass industry production of spalted wood products. That spalting pigments carried in DCM do not color the inside of wood. This is problematic because woodworkers tend to apply the coloration and then proceed with the finishing (turning, sanding and varnishing) of their products. Hence, during the final sanding procedure, the spalting pigments vanish.

This paper explores alternative carrier solvents for their use with resolubilized fungal dyes from *Chlorociboria aeruginosa* and *Scytalidium cuboideum*, in order to find an effective substitute for DCM that not only has the ability to carry the fungal pigments, but also to decrease the solubility of the pigment, so that color is left on the inside of wood. The solvents selected for this experiment were previously tested along with DCM but were not effective at extracting the fungal pigments from wood chips. Their use as carriers of resolubilized pigments (instead of extraction agents and carriers) might allow for increased internal pigmentation. In order to potentially commercialize spalted wood, and to broadly appeal to hobbyist and professional woodworkers, the issues related to colorant application and internal pigmentation must be addressed, or spalted wood will remain a woodworking curiosity, and a controlled spalting process will only be achievable in the laboratory.

## 2. Materials and Methods

Thirteen wood species, common to the Pacific Northwest of the USA, and previously utilized in spalting experiments, were selected [25,26], and the wood was cut into cubes (14 mm^3^) and air dried. Wood species used were: ash (*Fraxinus latifolia* Benth.), chinkapin (*Castanea pumila* Mill.), Douglas-fir (*Pseudotsuga menziesii* (Mirb.) Franco), lodgepole pine (*Pinus contorta* Douglas), madrone (*Arbutus menziesii* Pursh), mountain hemlock (*Tsuga mertensiana* (Bong.) Carr.), Oregon maple (*Acer macrophyllum* Pursh), pacific silver fir (*Abies amabilis* Douglas ex. J Forbes), Port Orford cedar (*Chamaecyparis lawsoniana* (A. Murray) Parl.), red alder (*Alnus rubra* Bong.), sugar maple (*Acer saccharum* Marshall), western larch (*Larix occidentalis* Nutt.), and western red cedar (*Thuja plicata* Donn ex D. Don).

Two fungal species were selected: *Scytalidium cuboideum* (Sacc. & Ellis) Sigler & Kang UAMH 4802 (isolated from red oak lumber, location unknown) and *Chlorociboria aeruginosa* (Nyl.) Kanouse UAMH 7615 (isolated from a single ascospore, in Lake District, UK). Both fungal species are known to produce pigments and have been previously studied for their spalting potential [9,24,35,38].

Fungal cultures were plated and then grown on a sterile mixture of 2% MEA (20 gr/L of malt extract, VWR, Radnor, PA, USA) and 15 gr/L of bacteriological agar (VWR)) and white rotted wood chips (*Acer saccharum*) for a month, via the procedure in Robinson, 2012 [9]. Pigments were extracted using dichloromethane using the protocol noted in Robinson, 2014 [35], and then color read with a Konica Minolta Chroma Meter CR-5 color reader. The data were collected with the Spectramagic NX, Color Data Software (Ver 2.8, Konica Minolta, Ramsey, NJ, USA), in order to meet specification standards determined by conversion from rough pigment weight to L*a*b* values [26].

The L*a*b* standards are as follows [26], and the values of the vials must fall +/−2.0 of these standards:

*C. aeruginosa*: L* = 82.28, a* = −11.06, b* = −5.40

*S. cuboideum*: L* = 82.32, a* = 26.84, b* = 13.19

Eight dram clear glass vials (ACE Glass Inc, Nineland, NJ, USA) containing the extracted pigments carried in DCM were used to evaporate the solvent using a Büchi rotovap (BÜCHI Labortechnik, New Castle, DE, USA), model 461, in a cold water bath, until only dry pigment remained in the vials. Then, the pigments were resolubilized using five different solvents: acetone, acetonitrile (ACN), chloroform, pyridine, and tetrahydrofuran (THF), utilizing the method determined in Robinson, 2014 [35].

The vials of pigment solution were placed on a Thermo Scientific LabQuake shaker (400110Q, ThermoFisher Scientific, Pittsburgh, PA, USA) for 24–48 h at room temperature (20 °C). This time was chosen as a uniform length for rotation, in order to give pigments time to solubilize.

Four drip treatments were performed with each solvent on the wood blocks (three blocks per wood type per treatment) using borosilicate glass disposable Pasteur pipettes (14673-010, VWR, Radnor, PA, USA), with reusable VWR latex pipet bulbs (82024-550, VWR, Radnor, PA, USA). Drips were on the transverse face. Drips were calibrated to the standard in Robinson, 2014 [26], with the average volume of a drop at 0.0165 mL.

Dripping treatments performed were: 16 drops (8 drops, wait 24 h, 8 drops), 30 drops (15, wait 24 h, 15 drops), 30 drops (applied in one session without waiting), and 60 drops (30 drops, wait 24 h, 30 drops). These drip treatments were performed for all solvents except for pyridine, which only had the first three performed (16, 30 drops straight, and 30 drops with waiting), as it became too viscous to allow more drops to enter the wood after 30 drops. The blocks were left in a fume hood for 24 h following the drip treatment. This process was performed with both *C. aeruginosa* (green) and *S. cuboideum* (red) pigments.

After drying, each block was cut in half along the radial face with a Grizzly bandsaw (61.75” blade) (Grizzly Industrial, Bellingham, WA, USA). The interior of one half of each block was scanned using an Epson Perfection V370 Photo scanner (Epson America Inc., Long Beach, CA, USA), and Epson Scan software (v3.9.4.1, Epson America Inc., Long Beach, CA, USA). Each scan was color evaluated using the protocol for ImageJ (v1.49, NIH, Bethesda, MD, USA) by Robinson et al. [39]. Three-way ANOVA tests followed by Tukey HSD (honest significant difference) were run for each wood species, with percent coverage as the dependent variable, and color; solvent; and drops as independent variables.

## 3. Results

Results for the 3-way ANOVA were significant for ash, chinkapin, Douglas-fir, madrone, mountain hemlock, Oregon maple, Port Orford cedar, pacific silver fir, red alder, and sugar maple, all with a *p*-value < 0.0001. Color, solvent, and drops were all significant for this group of wood species at *p* < 0.0001.

Western larch had no internal pigment so was not used in statistical analysis. Lodgepole pine and western red cedar had no significant results in 3-way ANOVA, 2-way ANOVA, and 1-way ANOVA tests.

### 3.1. Ash

The two treatments that were most effective for ash were *S. cuboideum* with chloroform and 30 straight drops, as well as with acetone with 16 drops. However, these treatments were not significantly different from: *S. cuboideum* with acetonitrile with 16 drops, both treatments with *C. aeruginosa* with acetone and 30 drops (30 with waiting 24 h and 30 straight), *S. cuboideum* with acetonitrile and 30 drops with waiting, *S. cuboideum* with tetrahydrofuran and 30 straight drops, *C. aeruginosa* with acetonitrile and 30 straight drops, *C. aeruginosa* with acetonitrile and 16 drops, and *C. aeruginosa* with acetone and 60 drops. Please refer to Supplementary Materials Table S1 for more detailed information.

### 3.2. Chinkapin

**Figure 2:**
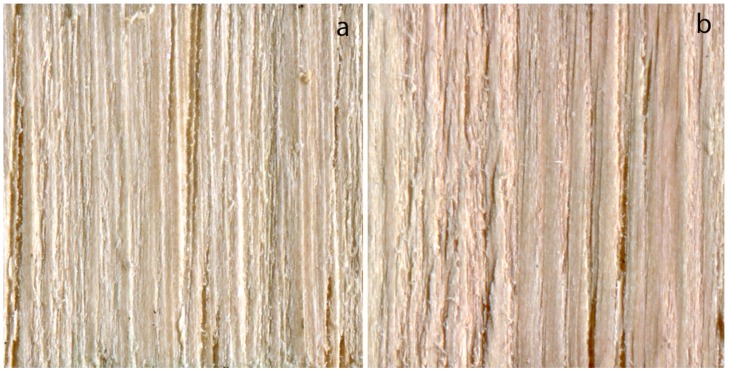
(**a**) Chinkapin control; (**b**) *Scytalidium cuboideum* pigment resolubilized in acetone (60 drops treatment).

The most effective treatment for chinkapin was *S. cuboideum* with acetone and 60 drops. This treatment had a 100% coverage average as seen in Figure 2. However, it did not differ significantly from many others, as seen in Table 1. No treatments with *C. aeruginosa* performed well.

### 3.3. Douglas-Fir, Pacific Silver Fir and Red Alder

The most effective treatments for Douglas-fir were *S. cuboideum*, one with pyridine and 16 drops and the other with acetonitrile and 30 straight drops. These two treatments were determined to be significantly different compared to every other treatment performed on Douglas-fir. Similar results were observed for Pacific silver fir and red alder, as the most effective treatment was *S. cuboideum* with acetonitrile and 30 drops with waiting for both species. The most effective treatment for red alder was *S. cuboideum* with acetonitrile and 30 drops with waiting. Please refer to Supplementary Materials Tables S2–S4 for more detailed information.

### 3.4. Madrone

**Figure 3:**
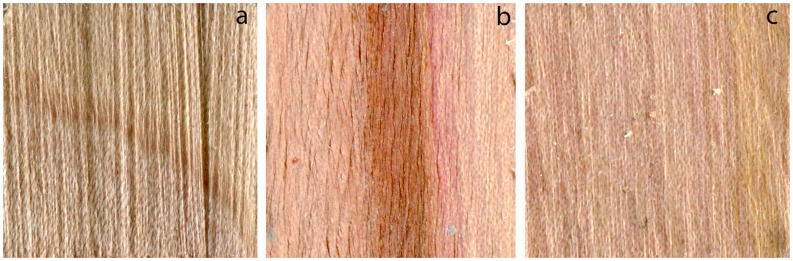
(**a**) Control for madrone’s internal coloration; (**b**) *Scytalidium cuboideum* pigment resolubilized in acetone (60 drops); (**c**) *Scytalidium cuboideum* pigment resolubilized in acetonitrile (30 drops with waiting).

Many of the treatments with madrone were statistically significant, as seen in Table 2. All of the statistically significant treatments had a mean percent coverage of 85% or higher, with the two highest means being 100% coverage (*S. cuboideum* with acetone and 60 drops, *S. cuboideum* with acetonitrile and 30 drops with waiting visualized in Figure 3). However, these treatments were not determined to be significantly different from many others.

### 3.5. Mountain Hemlock and Sugar Maple

The most effective treatment for mountain hemlock was *S. cuboideum* with acetonitrile and 30 straight drops, although it was not significantly different from several other treatments. Similar results for acetonitrile were obtained for sugar maple. For this wood species there was no significant difference between the 30 straight drops and *S. cuboideum* with pyridine and 16 drops and *S. cuboideum* with pyridine and 30 drops with waiting. No treatment with *C. aeruginosa* had over 3.667 mean percent coverage. Please refer to Supplementary Materials Tables S5 and S6 for more detailed information.

### 3.6. Oregon Maple and Port-Orford Cedar

The treatment that was most effective for Oregon maple was *S. cuboideum* with tetrahydrofuran and 30 straight drops, however the results were not different from *S. cuboideum* with acetonitrile and 30 drops with waiting. This was the only wood species where tetrahydrofuran was in the most effective treatment group. Similar results were obtained for Port-Orford cedar with *S. cuboideum* with acetonitrile and 30 straight drops. The results were not different from *S. cuboideum* with acetonitrile and 30 drops with waiting and *S. cuboideum* with tetrahydrofuran and 30 straight drops. Please refer to Supplementary Materials Tables S7 and S8 for more detailed information.

### 3.7. Western Larch, Lodgepole Pine, and Western Red Cedar

None of these treatments had significant results: none of the solvents were effective in depositing the fungal pigments in these wood species, and no internal color change was observed.

## 4. Discussion

In the analysis of the experiment data, acetonitrile combinations exhibited most interior coloration. Seven wood species (Douglas-fir, madrone, mountain hemlock, Port Orford cedar, pacific silver fir, red alder, and sugar maple) had an acetonitrile combination in the most statistically significant and effective grouping for all of the treatments. Ten treatments in the collection of effective combinations used acetonitrile. The second most effective solvent was acetone, as it was effective in four wood species (ash, chinkapin, Douglas-fir, and madrone), with eight treatments overall in that group. This indicates that acetonitrile, and to a lesser extent, acetone, are the most effective solvents for depositing pigments inside wood and are highly compatible with the red pigment from *S. cuboideum*. The anatomical features of the wood species could have also influenced the retention of the pigments, but this has been previously dealt with by Robinson et al. 2014 [25,26].

Out of the best performing solvents and across all the wood species, only madrone showed internal coloring from *C. aeruginosa*. That *C. aeruginosa* did not perform well with other solvents is not surprising, as it is notoriously difficult to solubilize from wood and is not stable in many solvents [35]. The pigment from *S. cuboideum*, in contrast, is much more stable and shows some affinity to solvents like THF and ACN. The difficulty of solubilizing the pigment from *C. aeruginosa* was one of the driving factors in the initial study on how to apply it to wood [25,26]. While it is disappointing that xylindein cannot be extracted with DCM and then carried into wood in a different solvent with which it has less affinity, the reasons for this are fairly straightforward. Xylindein has a strong affinity to DCM, and once the solvent evaporates from the solution, the pigment tends to attach to the surface where it has dried (for example, on the glass surface of the vials used to contain the DCM and pigment). The authors theorize that this is due to the available hydroxyl (–OH) sections in the xylindein molecule that can generate hydrogen bonds. These bonds are strong enough to avoid resolubilization in less polar solvents [40,41] as xylindein will also tend to bind to itself [17]. It is important to mention that the morphology of the pigments can also affect their capability to be carried by different solvents. In the case of xylindein, it has been determined that after extraction, the pigment has an amorphous structure when dry [21]. This type of configuration can increase the difficulty of the potential carriers to interact with active bonding areas for resolubilization.

For the pigment from *S. cuboideum*, the morphology has been observed as a suspected crystal [21], that has a more structured configuration, allowing solvents to interact with active areas for resolubilization in polar solvents. The morphology can also allow lower polarity solvents to interact with the molecule, allowing a wider variety of potential carriers. The chemical configuration of draconin red [20,21,22] in contrast with the one of xylindein [17] can affect the way that each pigment behaves in different solvents [35]. As observed within the experiment and previous studies, draconin red is soluble in a wider array of solvents, compared to xylindein. This difference could be due to the amount of hydroxyl (–OH) groups on each molecule available to interact with the solvents.

Acetonitrile (CH_3_CN) is a dipolar aprotic solvent. Though it has a larger dipole moment (3.90 D) than dichloromethane (1.60 D) [41] and has a higher polarity (18.0 δP compared to 7.3 δP), is possible that this resulted in a difference within the bonding capacity between acetonitrile and dichloromethane (6.1 δH compared to 7.1 δH). This difference was suggested in Robinson et al., 2014 [35], as effective extraction may be more dependent on molecular hydrogen bonding than dipole and polarity interactions [35], but further research should be performed to qualify and/or quantify the bonding type that occurs between the solvents and the pigments. Ranked from least polar to most polar, the solvents are: Tetrahydrofuran (THF), chloroform, acetone, pyridine, acetonitrile [42,43]. The polarity and solvent properties are most likely a contributing factor to the success of the solvent as a pigment deposition medium, as their capability for solubilizing molecules will vary according to the configuration of the molecules and their affinity to interact with the solvents. This factor can be seen in the results, as acetonitrile (the solvent with the highest polarity used in the experiment) performed the best with the fungal pigments with most of the wood species used, meanwhile the other solvents had inconsistent results. Another factor that could be involved is the difference in boiling points of the solvents. Dichloromethane has a boiling point of 39.5 °C [44], giving it the lowest boiling point of the solvents used. A lower boiling point generally implies a faster rate of evaporation under room temperature. Fast evaporation has been related to the inability of dichloromethane to successfully deposit the fungal pigments in wood [26]. In comparison, acetonitrile has a boiling point of 81.5 °C [45], giving it the second highest boiling point of solvents tested, just behind pyridine (115 °C) [46]. It is possible that there is an optimal solvent boiling temperature for the internal pigment deposit, since the higher boiling point of acetonitrile appears to work better for the internal deposition of fungal pigments. This is in contrast to the low boiling point of DCM, and its related poor internal pigment deposition. Other factors are likely at play, however, since pyridine has a higher boiling temperature than acetonitrile, it still created significant internal pigment due to its slower evaporation rate.

From the previously mentioned solvents, acetonitrile is a considerably cheaper and less toxic solvent than dichloromethane. It has been classified as Group D by the EPA (United States Environmental Protection Agency) [47], not classifiable to human carcinogenicity. This increases the possibility that it can be used in commercial dyes, due to the lower toxicity threat. Because acetonitrile holds onto pigment molecules less tightly (due to reduced ability to hydrogen bond) but has a high polarity, it may be able to hold onto pigment molecules (often high-molecular-weight polyaromatics) sufficiently for it to enter in the wood and deposit the pigment but not pull them through entirely. This deposits the dye molecules inside the piece of wood, rather than on the surface.

Recent research into oils as potential fungal pigment carriers has been performed with success, but the testing performed generally focused on their carrying capacity, polymerizing effects [33,48] and their applications in textiles [30]. As no testing has been performed with oils and wood, future research will need to compare the efficiency of acetonitrile and oils to internally deposit pigments in wood. It is also worth mentioning that for the preparation of the oils with fungal pigments, DCM is still required, as oils themselves are not well suited for direct extraction. The oil retains the pigment once that the DCM evaporates from the mixture [33,48] although it is possible that traces of DCM remain in the mixture. Further research within this topic needs to address a method by which these colorants could be utilized without caustic solvents, however for right now, the use of acetonitrile shows promising results. Acetonitrile is especially useful for home projects, as consumers would not be extracting their own pigment, instead purchasing it from a company that would complete the extraction process. Dichloromethane would still be used for extraction processes in the company or lab facilities and pigment could then be converted to dry pigment and resolubilized with acetonitrile for the customer to purchase and use. This would increase the marketability of spalted dyes overall and allow them to more fully compete within the woodworking aniline dye market.

## 5. Conclusions

Dichloromethane is still the best solvent for extracting spalting fungal pigments but acetonitrile may be a better candidate for resolubilization and application in art and industry, as it deposits pigments more effectively into wood. The selection of acetonitrile as a resolubilization carrier will also allow a wider use of the fungal pigments by woodworkers and industries interested in their use, because it is a solvent that is user friendly, has wider availability and lower cost. This can also allow the use of spalting fungal pigments in a wider variety of dye industries, making them a potential replacement for non-renewable synthetic dyes.

## Figures and Tables

**Table 1:** Statistical analysis results for chinkapin. Different letters under Tukey group represent significant differences at alpha = 0.05. The treatments applied were: 16w (8 drops, 24 h wait, 8 drops), 30w (15 drops, 24 h wait, 15 drops), 30 (30 drops with no wait) and 60w (30 drops, 24 h wait, 30 drops).

Wood	Solvent	Treatment	Pigment	Mean Percent Coverage	Standard Deviation
Chinkapin	Acetone	16w	Red	27 (CDEFG)	10.25
			Green	6.67 (EFG)	2.01
		30w	Red	26.67 (CDEFG)	9.63
			Green	0 (G)	0
		30	Red	92.33 (ABC)	29.35
			Green	0 (G)	0
		60w	Red	100 (A)	32.76
			Green	8.33 (EFG)	3.14
	Acetonitrile	16w	Red	26.33 (CDEFG)	8.82
			Green	0 (G)	0
		30w	Red	72.33 (ABCDE)	23.8
			Green	0 (G)	0
		30	Red	98.67 (AB)	31.17
			Green	0 (G)	0
		60w	Red	62.67 (ABCDEFG)	19.7
			Green	0 (G)	0
	Chloroform	16w	Red	1 (FG)	0.5
			Green	11.67 (EFG)	4.21
		30w	Red	24.33 (CDEFG)	7.74
			Green	23 (CDEFG)	6.49
		30	Red	1.33 (FG)	0.5
			Green	7.67 (EFG)	2.67
		60w	Red	60 (ABCDEFG)	18.15
			Green	3 (FG)	0.96
	THF	16w	Red	38.33 (ABCDEFG)	12.88
			Green	0 (G)	0
		30w	Red	65.67 (ABCDEFG)	21.16
			Green	0 (G)	0
		30	Red	59 (ABCDEFG)	16.42
			Green	28.67 (CDEFG)	10.85
		60w	Red	85 (ABCD)	25.75
			Green	10 (EFG)	3.67
	Pyridine	16w	Red	89.33 (ABCD)	27.62
			Green	13.33 (EFG)	4.78
		30w	Red	67 (ABCDEF)	22.43
			Green	29.33 (BCDEFG)	11.36
		30	Red	46.67 (ABCDEFG)	14.26
			Green	32.67 (BCDEFG)	11.98
		60w	Red	-	-
			Green	-	-

**Table 2:** Statistical analysis results for madrone. Different letters under Tukey group represent significant differences at alpha = 0.05. The treatments applied were: 16w (8 drops, 24 h wait, 8 drops), 30w (15 drops, 24 h wait, 15 drops), 30 (30 drops with no wait) and 60w (30 drops, 24 h wait, 30 drops).

Wood	Solvent	Treatments	Pigment	Mean Percent Coverage	Standard Deviation
Madrone	Acetone	16w	Red	88.33 (A)	62.46
			Green	93.33 (A)	65.99
		30w	Red	20 (BC)	14.14
			Green	53.33 (ABC)	37.71
		30	Red	90 (A)	63.64
			Green	40 (ABC)	28.28
		60w	Red	100 (A)	70.71
			Green	96.67 (A)	68.36
	Acetonitrile	16w	Red	68.33 (AB)	48.32
			Green	96.67 (A)	68.36
		30w	Red	100 (A)	70.71
			Green	86.67 (A)	61.28
		30	Red	85 (A)	60.1
			Green	83.33 (AB)	58.92
		60w	Red	60 (ABC)	42.43
			Green	43.33 (ABC)	30.64
	Chloroform	16w	Red	0 (C)	0
			Green	0 (C)	0
		30w	Red	86.67 (A)	61.28
			Green	0 (C)	0
		30	Red	93.33 (A)	65.99
			Green	0 (C)	0
		60w	Red	53.33 (ABC)	37.71
			Green	0 (C)	0
	THF	16w	Red	0 (C)	0
			Green	0 (C)	0
		30w	Red	0 (C)	0
			Green	0 (C)	0
		30	Red	0 (C)	0
			Green	0 (C)	0
		60w	Red	0 (C)	-
			Green	0 (C)	0
	Pyridine	16w	Red	83.33 (AB)	58.92
			Green	0 (C)	0
		30w	Red	0 (C)	0
			Green	0 (C)	0
		30	Red	85 (A)	60.1
			Green	0 (C)	0
		60w	Red	-	-
			Green	-	-

## Supplementary Materials

The following are available online at http://www.mdpi.com/1996-1944/11/6/897/s1, Table S1: Statistical analysis results for ash. Different letters under Tukey group represent significant differences at alpha = 0.05. The treatments applied were: 16w (8 drops, 24 h wait, 8 drops), 30w (15 drops, 24 h wait, 15 drops), 30 (30 drops with no wait) and 60w (30 drops, 24 h wait, 30 drops). Table S2: Statistical analysis results for Douglas-fir. Different letters under Tukey group represent significant differences at alpha = 0.05. The treatments applied were: 16w (8 drops, 24 h wait, 8 drops), 30w (15 drops, 24 h wait, 15 drops), 30 (30 drops with no wait) and 60w (30 drops, 24 h wait, 30 drops), Table S3: Statistical analysis results for mountain hemlock. Different letters under Tukey group represent significant differences at alpha = 0.05. The treatments applied were: 16w (8 drops, 24 h wait, 8 drops), 30w (15 drops, 24 h wait, 15 drops), 30 (30 drops with no wait) and 60w (30 drops, 24 h wait, 30 drops). Table S4: Statistical analysis results for Oregon maple. Different letters under Tukey group represent significant differences at alpha = 0.05. The treatments applied were: 16w (8 drops, 24 h wait, 8 drops), 30w (15 drops, 24 h wait, 15 drops), 30 (30 drops with no wait) and 60w (30 drops, 24 h wait, 30 drops). Table S5: Statistical analysis results for Port-Orford cedar. Different letters under Tukey group represent significant differences at alpha = 0.05. The treatments applied were: 16w (8 drops, 24 h wait, 8 drops), 30w (15 drops, 24 h wait, 15 drops), 30 (30 drops with no wait) and 60w (30 drops, 24 h wait, 30 drops). Table S6: Statistical analysis results for Pacific silver fir. Different letters under Tukey group represent significant differences at alpha = 0.05. The treatments applied were: 16w (8 drops, 24 h wait, 8 drops), 30w (15 drops, 24 h wait, 15 drops), 30 (30 drops with no wait) and 60w (30 drops, 24 h wait, 30 drops). Table S7: Statistical analysis results for red alder. Different letters under Tukey group represent significant differences at alpha = 0.05. The treatments applied were: 16w (8 drops, 24 h wait, 8 drops), 30w (15 drops, 24 h wait, 15 drops), 30 (30 drops with no wait) and 60w (30 drops, 24 h wait, 30 drops). Table S8: Statistical analysis results for sugar maple. Different letters under Tukey group represent significant differences at alpha = 0.05. The treatments applied were: 16w (8 drops, 24 h wait, 8 drops), 30w (15 drops, 24 h wait, 15 drops), 30 (30 drops with no wait) and 60w (30 drops, 24 h wait, 30 drops).
