# Supplementary material for: Alternative Carrier Solvents for Pigments Extracted from Spalting Fungi
Source: Materials (Basel). 2018 May 27;11(6):897. doi: 10.3390/ma11060897 (PMC6025569; doi:10.3390/ma11060897)
Supplement: Supplementary file 1 [file materials-11-00897-s001.pdf]

Supplementary Material

Table S1. Statistical analysis results for ash. Different letters under Tukey group represent significant differences at alpha = 0.05. The treatments applied were: 16w (8 drops, 24 h wait, 8 drops), 30w (15 drops, 24 h wait, 15 drops), 30 (30 drops with no wait) and 60w (30 drops, 24 h wait, 30 drops).

Wood	Solvent	Test	Pigment	Mean percent coverage	Standard deviation
Ash	Acetone	16w	Red	59.33 (A)	14.71
			Green	0 (B)	0.00
		30w	Red	1.33 (B)	0.45
			Green	35 (AB)	10.29
		30	Red	0 (B)	0.00
			Green	33.67 (AB)	9.11
		60w	Red	0 (B)	0.00
			Green	16 (AB)	3.67
	Acetonitrile	16w	Red	46 (AB)	12.12
			Green	19 (AB)	4.76
		30w	Red	30 (AB)	7.72
			Green	8.33 (B)	2.13
		30	Red	0 (B)	0.00
			Green	20 (AB)	6.30
		60w	Red	8.67 (B)	2.53
			Green	0 (B)	0.00
	Chloroform	16w	Red	1.33 (B)	0.38
			Green	0.33 (B)	0.08
		30w	Red	0.67 (B)	0.17
			Green	4 (B)	1.26
		30	Red	61 (A)	16.81
			Green	0 (B)	0.00
		60w	Red	6 (B)	1.61
			Green	0 (B)	0.00
	THF	16w	Red	0 (B)	0.00
			Green	0 (B)	0.00

		30w	Red	0 (B)	0.00
			Green	0 (B)	0.00
		30	Red	20 (AB)	5.64
			Green	0 (B)	0.00
		60w	Red	0 (B)	------
			Green	1.33 (B)	0.50
	Pyridine	16w	Red	4 (B)	1.07
			Green	3.33 (B)	0.80
		30w	Red	0 (B)	0.00
			Green	0 (B)	0.00
		30	Red	0 (B)	0.00
			Green	1 (B)	0.27
		60w	Red	-	-
			Green	-	-

Table S2. Statistical analysis results for Douglas-fir. Different letters under Tukey group represent significant differences at alpha = 0.05. The treatments applied were: 16w (8 drops, 24 h wait, 8 drops), 30w (15 drops, 24 h wait, 15 drops), 30 (30 drops with no wait) and 60w (30 drops, 24 h wait, 30 drops)

**Wood**	**Solvent**	**Test**	**Pigment**	**Mean percent coverage**	**Standard deviation**
Douglas-fir	Acetone	16w	Red	0 (C)	0
			Green	0.33 (C)	0.24
		30w	Red	0 (C)	0
			Green	0 (C)	0
		30	Red	0 (C)	0
			Green	0 (C)	0
		60w	Red	0 (C)	0
			Green	0 (C)	0
	Acetonitrile	16w	Red	0 (C)	0
			Green	0 (C)	0
		30w	Red	4 (BC)	2.83
			Green	0 (C)	0
		30	Red	19.67 (A)	13.91
			Green	0 (C)	0

		60w	Red	0 (C)	0
			Green	0 (C)	0
	Chloroform	16w	Red	0 (C)	0
			Green	0 (C)	0
		30w	Red	0 (C)	0
			Green	0 (C)	0
		30	Red	0 (C)	0
			Green	0 (C)	0
		60w	Red	0 (C)	0
			Green	0 (C)	0
	THF	16w	Red	0 (C)	0
			Green	0 (C)	0
		30w	Red	0 (C)	0
			Green	0 (C)	0
		30	Red	10 (B)	7.07
			Green	0 (C)	0
		60w	Red	0 (C)	------
			Green	0 (C)	0
	Pyridine	16w	Red	25 (A)	17.68
			Green	7.67 (BC)	5.43
		30w	Red	5.33 (BC)	3.77
			Green	0 (C)	0
		30	Red	8.33 (BC)	5.89
			Green	0 (C)	0
		60w	Red	-	-
			Green	-	-

Table S3. Statistical analysis results for Pacific silver fir. Different letters under Tukey group represent significant differences at alpha = 0.05. The treatments applied were: 16w (8 drops, 24 h wait, 8 drops), 30w (15 drops, 24 h wait, 15 drops), 30 (30 drops with no wait) and 60w (30 drops, 24 h wait, 30 drops).

**Wood**	**Solvent**	**Test**	**Pigment**	**Mean percent coverage**	**Standard deviation**
	Acetone	16w	Red	0.67 (C)	0.22

			Green	6.33 (BC)	1.5
		30w	Red	3.67 (C)	1.11
			Green	0 (C)	0
		30	Red	0 (C)	0
			Green	0 (C)	0
		60w	Red	0 (C)	0
			Green	0 (C)	0
	Acetonitrile	16w	Red	2.33 (C)	0.69
			Green	0 (C)	0
		30w	Red	75.33 (A)	13.67
			Green	0 (C)	0
		30	Red	34.67 (B)	7.28
			Green	0 (C)	0
		60w	Red	0.33 (C)	0.11
			Green	0 (C)	0
Pacific silver fir	Chloroform	16w	Red	0 (C)	0
			Green	1.33 (C)	0.38
		30w	Red	0 (C)	-----
			Green	6.67 (BC)	2.02
		30	Red	0.33 (C)	0.083
			Green	2 (C)	0.58
		60w	Red	0 (C)	0
			Green	8.33 (BC)	2.34
	THF	16w	Red	0.67 (C)	0.19
			Green	0 (C)	0
		30w	Red	1 (C)	0.3
			Green	0 (C)	0
		30	Red	3.33 (C)	0.97
			Green	0 (C)	0
		60w	Red	1.67 (C)	0.48
			Green	0.67 (C)	0.25
	Pyridine	16w	Red	6.67 (BC)	1.8
			Green	10.33 (BC)	2.76
		30w	Red	13 (BC)	3.32

			Green	3 (C)	0.833
		30	Red	25.67 (BC)	5.11
			Green	C	0.128
		60w	Red	-	-
			Green	-	-

Table S4. Statistical analysis results for red alder. Different letters under Tukey group represent significant differences at alpha = 0.05. The treatments applied were: 16w (8 drops, 24 h wait, 8 drops), 30w (15 drops, 24 h wait, 15 drops), 30 (30 drops with no wait) and 60w (30 drops, 24 h wait, 30 drops).

Wood	Solvent	Test	Pigment	Mean percent coverage	Standard deviation
Red alder	Acetone	16w	Red	0 (C)	0
			Green	2.67 (C)	0.46
		30w	Red	0 (C)	0
			Green	0 (C)	0
		30	Red	0 (C)	0
			Green	0 (C)	0
		60w	Red	0 (C)	0
			Green	0 (C)	0
	Acetonitrile	16w	Red	10 (B)	2.29
			Green	0 (C)	0
		30w	Red	54.33 (A)	8.99
			Green	0 (C)	0
		30	Red	4.33 (BC)	0.85
			Green	0 (C)	0
		60w	Red	0 (C)	0
			Green	0 (C)	0
	Chloroform	16w	Red	0 (C)	0
			Green	0 (C)	0
		30w	Red	0 (C)	0
			Green	0 (C)	0
		30	Red	0.33 (C)	0.06
			Green	0 (C)	0
		60w	Red	0 (C)	0

			Green	0 (C)	0
	THF	16w	Red	0 (C)	0
			Green	0 (C)	0
		30w	Red	0 (C)	0
			Green	0 (C)	0
		30	Red	9 (B)	1.69
			Green	0 (C)	0
		60w	Red	0 (C)	-----
			Green	0 (C)	0
	Pyridine	16w	Red	0 (C)	0
			Green	0 (C)	0
		30w	Red	0 (C)	0
			Green	0 (C)	0
		30	Red	0 (C)	0
			Green	0 (C)	0
		60w	Red	-	-
			Green	-	-

Table S5. Statistical analysis results for mountain hemlock. Different letters under Tukey group represent significant differences at alpha = 0.05. The treatments applied were: 16w (8 drops, 24 h wait, 8 drops), 30w (15 drops, 24 h wait, 15 drops), 30 (30 drops with no wait) and 60w (30 drops, 24 h wait, 30 drops).

Wood	Solvent	Test	Pigment	Mean percent coverage	Standard deviation
Mountain hemlock	Acetone	16w	Red	B	0
			Green	2.33 (B)	0.58
		30w	Red	33 (AB)	8.91
			Green	0 (B)	0
		30	Red	0 (B)	0
			Green	0 (B)	0
		60w	Red	33.33 (AB)	9.96
			Green	1.33 (B)	0.41
	Acetonitrile	16w	Red	25.67 (AB)	6.58
			Green	0 (B)	0

		30w	Red	34.67 (AB)	10.9
			Green	0 (B)	0
		30	Red	58.33 (A)	13.63
			Green	0 (B)	0
		60w	Red	4.33 (B)	0.98
			Green	0.33 (B)	0.14
	Chloroform	16w	Red	0 (B)	0
			Green	0 (B)	0
		30w	Red	0 (B)	0
			Green	0.67 (B)	0.25
		30	Red	0.33 (B)	0.13
			Green	0 (B)	0
		60w	Red	0.33 (B)	0.11
			Green	0 (B)	0
	THF	16w	Red	0.67 (B)	0.19
			Green	4.33 (B)	1.22
		30w	Red	0.33 (B)	0.09
			Green	0 (B)	0
		30	Red	11.67 (B)	2.94
			Green	1.33 (B)	0.35
		60w	Red	6.67 (B)	1.66
			Green	0.67 (B)	0.23
	Pyridine	16w	Red	21.33 (AB)	5.34
			Green	9 (B)	2.24
		30w	Red	14.33 (AB)	4.22
			Green	26.67 (AB)	7.59
		30	Red	0 (B)	-----
			Green	13.67 (AB)	3.64
		60w	Red	-	-
			Green	-	-

Table S6. Statistical analysis results for sugar maple. Different letters under Tukey group represent significant differences at alpha = 0.05. The treatments applied were: 16w (8 drops, 24 h wait, 8 drops), 30w (15 drops, 24 h wait, 15 drops), 30 (30 drops with no wait) and 60w (30 drops, 24 h wait, 30 drops).

Wood	Solvent	Test	Pigment	Mean percent coverage	Standard deviation
Sugar maple	Acetone	16w	Red	0 (B)	0
			Green	0 (B)	0
		30w	Red	7 (B)	2.16
			Green	0 (B)	0
		30	Red	0 (B)	0
			Green	0 (B)	0
		60w	Red	1 (B)	0.34
			Green	0 (B)	0
	Acetonitrile	16w	Red	1.67 (B)	0.45
			Green	0 (B)	0
		30w	Red	24.67 (A)	4.93
			Green	0 (B)	0
		30	Red	4.33 (B)	1.33
			Green	0 (B)	0
		60w	Red	2.33 (B)	0.61
			Green	0 (B)	0
	Chloroform	16w	Red	1 (B)	0.22
			Green	0 (B)	0
		30w	Red	0 (B)	-----
			Green	3.7 (B)	1.17
		30	Red	0 (B)	0
			Green	0 (B)	0
		60w	Red	1 (B)	0.29
			Green	0 (B)	0
	THF	16w	Red	9 (B)	2.49
			Green	0 (B)	0
		30w	Red	5.33 (B)	1.55
			Green	0 (B)	0
		30	Red	6.33 (B)	1.95
			Green	0 (B)	0
		60w	Red	3.67 (B)	1.03
			Green	0 (B)	0

	Pyridine	16w	Red	12.33 (AB)	3.35
			Green	0 (B)	0
		30w	Red	11 (AB)	2.91
			Green	0.33 (B)	0.07
		30	Red	5.67 (B)	1.74
			Green	3.33 (B)	0.84
		60w	Red	-	-
			Green	-	-

Table S7. Statistical analysis results for Oregon maple. Different letters under Tukey group represent significant differences at alpha = 0.05. The treatments applied were: 16w (8 drops, 24 h wait, 8 drops), 30w (15 drops, 24 h wait, 15 drops), 30 (30 drops with no wait) and 60w (30 drops, 24 h wait, 30 drops).

**Wood**	**Solvent**	**Test**	**Pigment**	**Mean percent coverage**	**Standard deviation**
Oregon maple	Acetone	16w	Red	0.67 (B)	0.18
			Green	4.67 (B)	1.49
		30w	Red	1.67 (B)	0.58
			Green	0 (B)	0
		30	Red	0 (B)	0
			Green	0 (B)	0
		60w	Red	0 (B)	0
			Green	0 (B)	0
	Acetonitrile	16w	Red	7.33 (B)	2.13
			Green	3.33 (B)	1.22
		30w	Red	28 (AB)	5.57
			Green	8.33 (B)	2.63
		30	Red	2.33 (B)	0.81
			Green	0 (B)	0
		60w	Red	1.33 (B)	0.35
			Green	0 (B)	0
	Chloroform	16w	Red	0 (B)	0
			Green	0 (B)	0
		30w	Red	0.33 (B)	0.08

			Green	12.33 (B)	3.78
		30	Red	1.67 (B)	0.49
			Green	4 (B)	1.34
		60w	Red	0 (B)	0
			Green	11.67 (B)	3.07
	THF	16w	Red	0 (B)	-----
			Green	6.67 (B)	1.88
		30w	Red	2.33 (B)	0.72
			Green	0 (B)	0
		30	Red	45 (A)	8.68
			Green	2.67 (B)	0.92
		60w	Red	3 (B)	1.03
			Green	0 (B)	0
	Pyridine	16w	Red	5 (B)	1.63
			Green	12.33 (B)	3.47
		30w	Red	0 (B)	0
			Green	3.33 (B)	1.14
		30	Red	0 (B)	0
			Green	8.33 (B)	2.41
		60w	Red	-	-
			Green	-	-

Table S8. Statistical analysis results for Port-Orford cedar. Different letters under Tukey group represent significant differences at alpha = 0.05. The treatments applied were: 16w (8 drops, 24 h wait, 8 drops), 30w (15 drops, 24 h wait, 15 drops), 30 (30 drops with no wait) and 60w (30 drops, 24 h wait, 30 drops).

**Wood**	**Solvent**	**Test**	**Pigment**	**Mean percent coverage**	**Standard deviation**
Port-Orford cedar	Acetone	16w	Red	23.67 (DEF)	8.06
			Green	0 (F)	0
		30w	Red	19 (DEF)	6.52
			Green	0 (F)	0
		30	Red	6 (EF)	1.97
			Green	0 (F)	0
		60w	Red	20.33 (DEF)	7.62

			Green	19.67 (DEF)	6.94
	Acetonitrile	16w	Red	38.33 (BCD)	10.99
			Green	0 (F)	0
		30w	Red	66.67 (AB)	16.55
			Green	0 (F)	0
		30	Red	72.33 (A)	18.94
			Green	0 (F)	0
		60w	Red	33.67 (CDE)	9.99
			Green	0 (F)	0
	Chloroform	16w	Red	14 (DEF)	4.92
			Green	11 (DEF)	3.83
		30w	Red	4.33 (EF)	1.17
			Green	12.33 (DEF)	4.52
		30	Red	30 (DEF)	9.16
			Green	1.67 (F)	0.5
		60w	Red	0 (F)	-----
			Green	8.67 (DEF)	2.88
	THF	16w	Red	7.33 (DEF)	1.55
			Green	0 (F)	0
		30w	Red	17.67 (DEF)	5.55
			Green	0 (F)	0
		30	Red	64 (ABC)	14.06
			Green	0 (F)	0
		60w	Red	4.67 (EF)	0
			Green	0 (F)	0.28
	Pyridine	16w	Red	18 (DEF)	6.05
			Green	20 (DEF)	7.3
		30w	Red	24 (DEF)	8.45
			Green	11.33 (DEF)	4.17
		30	Red	10.33 (DEF)	3.4
			Green	0 (F)	0
		60w	Red	-	-
			Green	-	-
